# Continuous Livestock Archetypes for Characterizing Structural Heterogeneity in Dual-Purpose Cattle Farms in the Ecuadorian Amazon

**DOI:** 10.3390/ani16182931

**Published:** 2026-09-18

**Authors:** Juan Urdánigo-Zambrano, Bolier Torres, Carmen De-Pablos-Heredero, Ana González-Martínez, Yenny Torres, Antón García

**Affiliations:** 1 Facultad de Ciencias Pecuarias, Universidad Técnica Estatal de Quevedo (UTEQ), Quevedo Av. Quito km, 1 ½ Vía a Santo Domingo de los Tsáchilas, Quevedo 120550, Ecuador; jurdanigo@uteq.edu.ec (J.U.-Z.); ytorres@uteq.edu.ec (Y.T.); 2 Natural Resources and Sustainable Management Program, Department of Animal Production, Faculty of Veterinary Sciences, University of Cordoba, 14071 Cordoba, Spain; 3 Departamento de Silvicultura y Producción Agrícola, Universidad Estatal Amazónica (UEA), Puyo 160101, Ecuador; btorres@uea.edu.ec; 4 Department of Business Economics (Administration, Management and Organization), Applied Economics II and Fundamentals of Economic Analysis, Rey Juan Carlos University, Paseo de los Artilleros s/n, 28032 Madrid, Spain; carmen.depablos@urjc.es; 5 Department of Animal Production, Faculty of Veterinary Sciences, University Cordoba, 14071 Cordoba, Spain; v32gomaa@uco.es

**Keywords:** farm heterogeneity, factor analysis of mixed data (FAMD), archetypal analysis, dual purpose cattle, sustainability, silvopastoral transition, Sumaco Biosphere Reserve

## Abstract

Farm typologies assume discrete groups, although structural heterogeneity in Amazonian cattle systems may follow continuous gradients. We evaluated whether 164 dual-purpose cattle farms in the Sumaco Biosphere Reserve, Ecuador, were better represented by mutually exclusive types or continuous archetypal configurations and whether these configurations were economically differentiated. Factor analysis of mixed data, permutation-based dimensional retention, clusterability assessment, archetypal analysis, bootstrap resampling, economic validation, and repeated cross-validation were integrated. Two structural dimensions explained 40.1% of inertia: livestock intensification and infrastructure, and farm scale and land use; agreement with multiple factor analysis was high (Procrustes *R*^2^ = 0.990). The Gap statistic supported *k* = 1, providing no evidence for defensible discrete farm groups. Archetypal analysis identified three continuous configurations: lower-input, livestock intensification, and large-scale land-use. Many farms occupied intermediate positions, showing varying affinities to the three configurations rather than exclusive membership in one. Greater affinity to livestock intensification was associated with the highest annual net benefit and pasture opportunity cost, whereas large-scale land-use showed intermediate economic performance. Predictive accuracy remained modest despite lower errors for archetype-based models. Continuous livestock archetypes provide a screening framework for differentiated management, but farm decisions require current economic, productive, and parcel information.

## Simple Summary

Cattle farms in the Ecuadorian Amazon differ considerably in farm area, herd size, forest cover, labor, and infrastructure, which makes it difficult to classify them into rigid farm types. We evaluated 164 dual-purpose cattle farms in the Sumaco Biosphere Reserve to determine whether they formed clearly separated groups or, instead, combined characteristics of different production strategies. The farms did not form well-defined groups. Instead, three reference configurations were identified: farms with low input levels and limited infrastructure, farms with greater livestock intensification and infrastructure, and larger farms with greater land and forest resources. Many farms occupied intermediate positions across the three configurations, showing different degrees of similarity to each one rather than belonging exclusively to a single configuration. Farms showing greater similarity to the livestock intensification configuration also tended to have better economic performance, whereas larger farms showed intermediate economic outcomes. Continuous configurations can help extension services, farmers, and conservation programs identify management options that are better suited to the structural conditions of each farm. However, they should be used as an initial guide rather than as permanent labels or as a substitute for a detailed assessment of each farm.

## 1. Introduction

Farm typologies synthesize the heterogeneity of agricultural systems into simplified representations that facilitate interpretation by researchers and decision-makers [1]. They help identify target groups for public policies, guide the delivery of technical assistance, and explain why uniform interventions are unlikely to meet the needs of farms with different resources and constraints [2]. Accordingly, typology development has increasingly been viewed as an iterative process encompassing construction, validation, application, and revision, rather than as a one-off clustering exercise [3]. This broader perspective is particularly relevant in forest–savannah transition zones, where farm structure and household decision-making should be considered jointly to support recommendations on land use and conservation [4]. More recently, generalized farm typologies have been proposed to improve the selection and scaling of on-farm research, extending the applicability of findings beyond the original study population [5].

The usual methodology of reducing dimensions and then imposing clusters is useful only when the observed data contains repeatable discontinuities [6,7]. Building on this established framework, the present study extends rather than replaces conventional farm typology approaches by explicitly evaluating whether discrete groups are empirically supported before adopting a continuous representation of farm heterogeneity. In contrast, archetypal analysis represents farm heterogeneity as continuous combinations of reference configurations instead of discrete farm classes [8]. Hypothesis-based frameworks similarly emphasize that the variables and the form of a typology must follow its intended use rather than an automatic statistical recipe [9]. Cluster validation research warns that an apparently clear solution in a development sample may not reproduce in resampled or validation data [10]. Evidence from mixed crop–livestock systems also indicates that domain specific profiles can provide more actionable targeting than one general hard partition [11]. This is particularly relevant in small-scale mixed crop–livestock systems, where farms frequently combine agricultural, livestock and forest-based activities, generating substantial internal heterogeneity that is difficult to represent through rigid farm classes and is better captured by continuous archetypal combinations [12]. These findings make the distinction between classes and continuous combinations a substantive question, not only a technical choice.

This distinction matters for land use decisions. Data-driven typologies have been proposed as a basis for agricultural land allocation [13], while comparative typology research has used farm trajectories to describe agroecological transitions [14]. Climate smart agriculture programs increasingly require methods that can scale targeting without erasing household diversity [15]. At the same time, household income diversification and partial withdrawal from agriculture can alter the meaning of structural farm categories [16]. Smallholder systems may contain productive potential that is not visible when low resource endowment is treated as a fixed state [17]. The century-long evolution of farming systems research therefore supports an integrated view in which farms are dynamic social, economic, and ecological units [18]. In this context, methodological approaches capable of representing farms as continuous combinations of production strategies, rather than as mutually exclusive categories, may provide a more realistic basis for supporting sustainable management and silvopastoral transitions in heterogeneous smallholder systems.

The Ecuadorian Amazon provides a relevant setting for examining farm heterogeneity. Previous studies characterized livestock production systems, identified management practices associated with forest conservation and the Sustainable Development Goals, and evaluated livelihood capitals and grazing opportunity costs from the predefined low-, middle-, and high-elevation zones of the Sumaco Biosphere Reserve [19,20]. Complementary research also highlighted the importance of silvopastoral systems, tree diversity, and ecosystem services in this region [21]. However, previous studies mainly characterized farm heterogeneity using predefined environmental or socioeconomic classifications. They did not test whether structural variation among farms forms reproducible discrete groups or is better represented by continuous combinations of structural configurations. This unresolved issue constitutes the main knowledge gap addressed in this study.

The research problem is therefore whether the structural heterogeneity of dual-purpose cattle farms in the Ecuadorian Amazon supports reproducible discrete farm types or is better represented by continuous archetypal configurations. This distinction is relevant because the chosen representation influences farm characterization, target-group identification, and the design of differentiated technical assistance and sustainability strategies. This issue is particularly important in the Ecuadorian Amazon, where livelihood capitals, agricultural diversification, and household strategies contribute to substantial heterogeneity among smallholder livestock systems [22,23].

Responsible reuse of existing datasets can also generate new scientific questions while improving analytical transparency and reproducibility [24]. Because farm structure and economic performance represent conceptually distinct dimensions of production systems, structural variables were used to characterize farm heterogeneity, whereas economic outcomes were reserved as independent criteria for subsequent validation [25].

For the purposes of hypothesis testing, a representation was considered more defensible when it was supported by convergent evidence from clusterability diagnostics, out-of-sample reconstruction performance, interpretability, and bootstrap reproducibility. We hypothesized that farm heterogeneity would be more appropriately represented by continuous archetypal configurations than by discrete farm types when reproducible group boundaries were not supported by the data. The study pursued three scientific, objectives: (1) to characterize the heterogeneity of dual-purpose cattle farms by jointly considering land use, herd structure, labor, infrastructure, and technological resources; (2) to identify continuous livestock archetypes representing the principal structural configurations of these farms; and (3) to validate the economic relevance of the resulting archetypes by examining their relationships with net benefit and pasture opportunity cost as independent validation criteria. The fourth objective was applied: (4) to develop a framework to inform differentiated management options based on farm affinity profiles, including technical assistance, ecological restoration, and potential silvopastoral transitions.

The analysis first tested whether structural variation supported reproducible discrete farm groups. Continuous archetypal configurations were then evaluated as an alternative representation of farm heterogeneity. Economic validation and out-of-sample prediction were used to assess the external relevance of the retained representation. The results supported interpreting farm heterogeneity as a continuum and using the archetypes to inform conditional management options. These management options were not experimentally tested. The framework is intended for dual-purpose cattle farmers, extension professionals, public decision-makers, and conservation organizations. Farm affinity profiles may help identify structural characteristics and prioritize farm-specific assessments. They may also improve the targeting of support programs, while recognizing that many farms occupy intermediate positions between reference configurations [26,27].

## 2. Materials and Methods

### 2.1. Study Area, Source Survey, and Analytical Positioning

**Figure 1:**
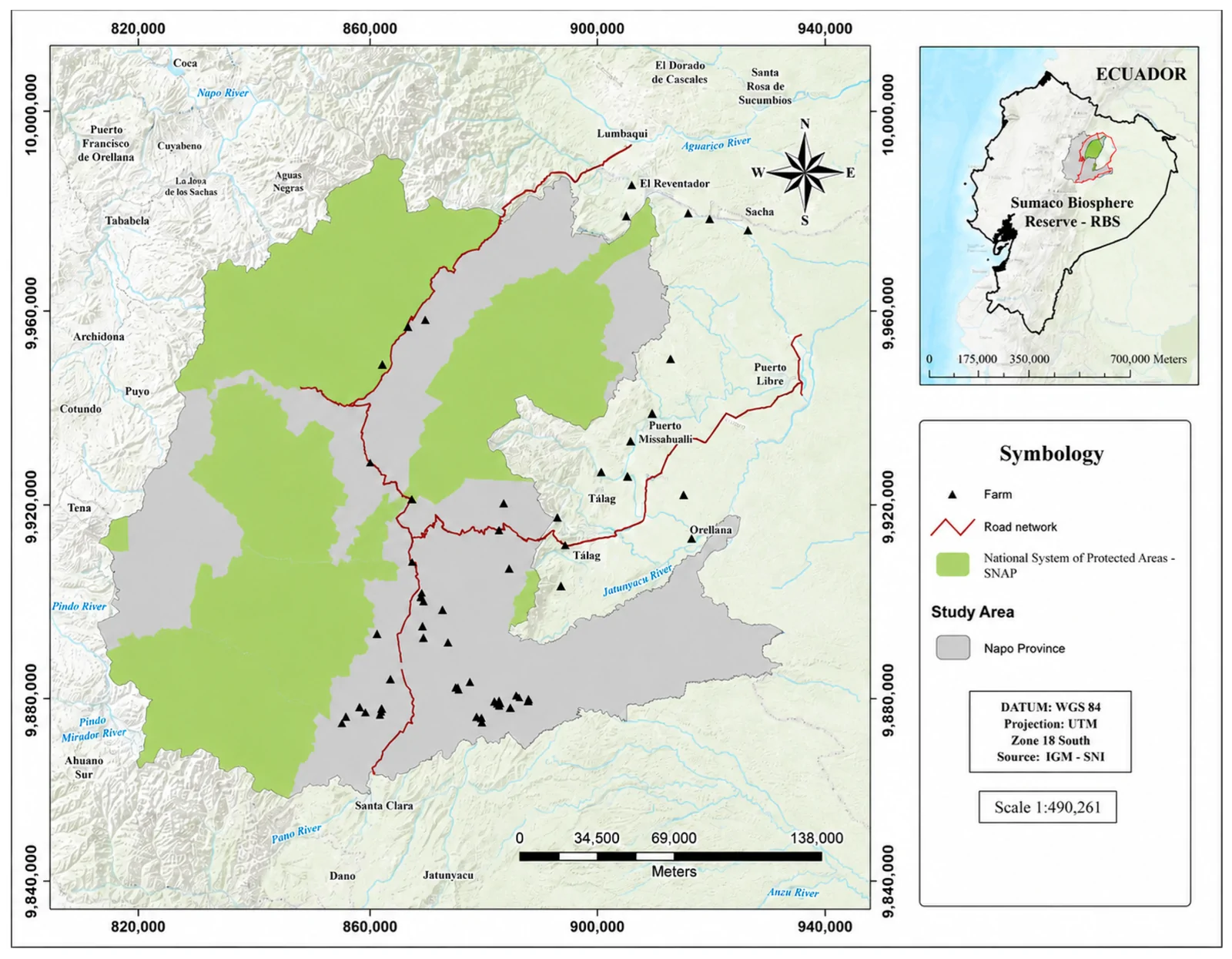
Location of the study area and sampled dual-purpose cattle farms in Napo Province, Ecuador.

An exploratory survey of cattle-farming systems in the study area was conducted in 2015. The principal cross-sectional household survey used in this study was conducted between 2021 and 2023 in the buffer and transition zones of the Sumaco Biosphere Reserve in the Ecuadorian Amazon (Figure 1). The sampling frame comprised 464 cattle-farming households distributed across three elevational zones within the Sumaco Biosphere Reserve. Stratified random sampling with proportional allocation across the low-, middle-, and high-elevation zones yielded 167 surveyed farms: 57 in the low zone, 57 in the middle zone, and 53 in the high zone. The surveyed farms operated dual-purpose cattle systems, producing both milk and meat, although the relative importance of these activities varied among individual farms. The farms were located in the cantons of Quijos (San Francisco de Borja), Archidona (Cotundo), and Carlos Julio Arosemena Tola.

For each farm surveyed, information was collected on land use, labor, herd structure, infrastructure, production costs, and income. Farms were eligible for the original survey if they had operated for at least three consecutive years and maintained more than 10 heads of cattle at the time of sampling. The present secondary analysis imposed no additional eligibility thresholds; all 167 available farm records entered the data-auditing stage. Structural information was derived from the 2021–2023 survey, whereas economic information was updated in 2025.

The sampling frame comprised 464 cattle-farming households distributed across three elevational zones within the Sumaco Biosphere Reserve. Stratified random sampling with proportional allocation across the low-, middle-, and high-elevation zones yielded 167 surveyed farms: 57 in the low zone, 57 in the middle zone, and 53 in the high zone. The surveyed farms operated dual-purpose cattle systems, producing both milk and meat, although the relative importance of these activities varied among farms and elevational zones. The farms were located in Quijos (San Francisco de Borja), Archidona (Cotundo), and Carlos Julio Arosemena Tola.

Information on land use, labor, herd structure, infrastructure, production costs, and income was collected through farm visits and in-person interviews. All 167 farm visits and interviews were conducted individually by the same interviewer; therefore, only one interviewer was present at each farm. Before fieldwork, the interviewer was trained by the study author responsible for custody of the original database. The training covered the objectives and scope of the study, eligibility criteria, questionnaire structure and sequence, operational definitions and variable coding, standardized and neutral administration of the questions, informed-consent procedures, confidentiality, and consistent data recording. The interviewer was also instructed to review each questionnaire for completeness and internal consistency before leaving the farm. No separate pilot test of the questionnaire was conducted.

Prior to data collection, preliminary meetings were held in the participating Indigenous communities to present the study and its general objectives. Selected producers were generally contacted through these community meetings, where subsequent farm visits were arranged. Farmers were informed in general terms about the purpose and scope of the survey, but they did not receive the questionnaire or individual questions in advance. The selected producers were subsequently approached through in-person farm visits. All selected producers were successfully located and agreed to participate; therefore, the refusal rate was 0%, there was no loss due to non-contact, and no replacements were required. Before each interview, the purpose and voluntary nature of participation were explained to the producer, and verbal informed consent was obtained.

Secondary analyses were performed using this dataset. Unlike previous studies, which organized analyses according to predefined altitudinal gradients [20,21], farm profiles were constructed without using elevation or any other environmental strata. Structural heterogeneity was analyzed to identify continuous livestock archetypes. Economic variables were reserved for independent validation and were excluded from archetype construction. The analytical strategy used for this secondary analysis is described in Section 2.2.

The audit checked unique identifiers, missingness, category codes, household and labor count coherence, duplication between raw and derived area fields, land component consistency, and outlying values, from the original database. Three records were excluded from the structural analysis as they combined multiple inconsistencies. The structural cohort therefore contained 164 farms.

The farm identified as record 8 in the source database was retained in the structural cohort because its land-use, livestock, labor, and infrastructure data were complete and internally coherent. However, it was excluded from the economic validation cohort because the total variable cost was missing, preventing the consistent calculation of annual net benefit. Consequently, the structural and economic cohorts comprised 164 and 163 farms, respectively. No active structural variable contained missing values in the final analytical dataset.

Variable selection combined statistical quality with livestock and land use relevance. The final representation comprised 11 active variables in three conceptual blocks. The land and land use block contained log-transformed total area, slope, crop area percentage, forest area percentage, and the percentage of pasture compatible with grazing. The herd and labor block contained log-transformed livestock inventory, counts of family members working on and off the farm, and the presence of hired labor. The infrastructure block contained infrastructure type, coded as none, rustic, or typical, and the presence of animal health and welfare housing conditions (Table 1).

Absolute and percentage measurements of the same land-use component were not included simultaneously in the active FAMD matrix to avoid redundancy and unintended double weighting. Total farm area was retained in hectares and log-transformed, whereas crop area, forest area, and grazing-compatible pasture were represented as percentages. Total farm area and livestock inventory were transformed as log (1 + x) to reduce the influence of right-skewed values while accommodating zeros.

### 2.2. Statistical Analysis

**Figure 2:**
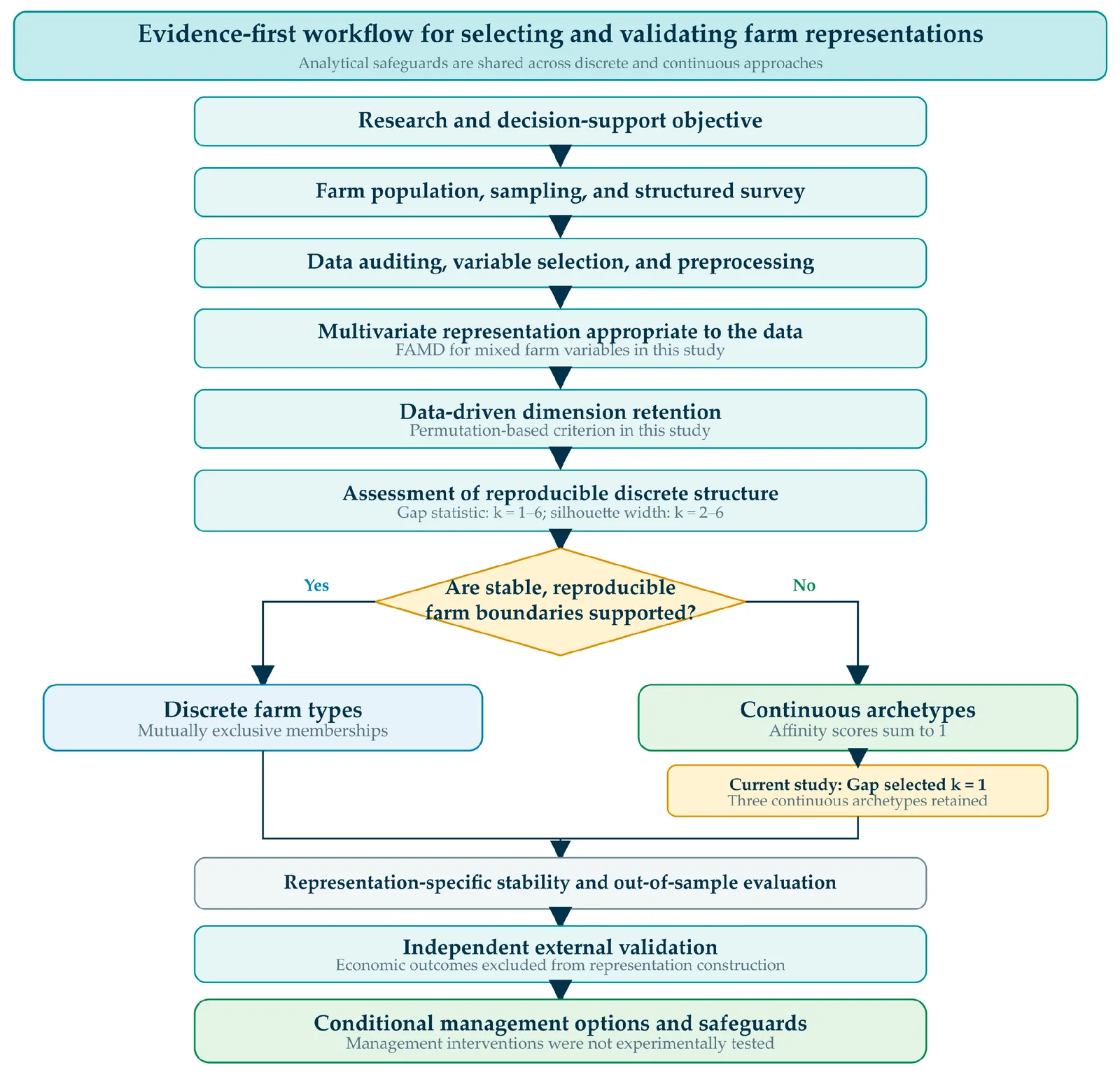
Evidence-first workflow for analyzing farm heterogeneity.

The statistical analysis followed five sequential stages: (1) construction and sensitivity assessment of the structural multivariate representation; (2) evaluation of evidence for discrete farm structure; (3) selection and stability assessment of continuous archetypes; (4) criterion-related economic validation; and (5) out-of-sample predictive evaluation. As summarized in Figure 2, data auditing, preprocessing, data-driven dimension retention, resampling, cross-validation, and criterion-related validation were treated as general analytical safeguards applicable to both discrete and continuous representations [9,10]. The distinctive feature of the workflow was the explicit assessment of reproducible discrete structure before selecting the final representation. Discrete farm types could be retained when stable boundaries were supported; otherwise, continuous archetypal affinities provided an alternative representation [8]. Economic outcomes excluded from representation construction were subsequently used as independent validation criteria [25].

First, Factor Analysis of Mixed Data (FAMD) was applied because the structural dataset contained both quantitative and qualitative variables [28]. Quantitative variables were centered and scaled, whereas qualitative variables were represented through indicator coding and category-frequency weighting consistent with FAMD geometry. Dimensionality was determined using 1000 permutation-based null datasets. An observed dimension was retained only when its eigenvalue exceeded the 95th percentile of the corresponding null distribution. Variable contributions were evaluated using 1000-bootstrap resamples. Variable influence on the retained representation was additionally assessed through permutation displacement. Each active variable was independently permuted across farms 1000 times while all other variables remained unchanged. Displacement was calculated as the mean squared difference between the original and permuted farm scores across the two retained standardized FAMD dimensions; larger values indicated greater influence on the structural representation. This variable-level measure is distinct from the archetype-center displacement used in the subsequent bootstrap stability analysis. As a sensitivity analysis, Multiple Factor Analysis was applied using land use, herd and labor, and infrastructure as separate conceptual blocks. Agreement between the FAMD and block-balanced representations was assessed using full Procrustes analysis, allowing translation, rotation or reflection, and isotropic scaling.

Second, evidence for discrete farm structure was evaluated before interpreting any partition as a set of farm types. The Gap statistic was calculated for solutions from (*k* = 1) to (*k* = 6), with the first-standard-error rule used as the primary criterion for selecting the number of groups [29]. Mean silhouette width was subsequently examined for forced partitions from (*k* = 2) to (*k* = 6) to assess their internal separation [30]. A discrete solution would be retained only if the Gap statistic supported (*k* > 1) and the corresponding partition showed adequate separation. Consequently, cluster solutions were treated as diagnostic alternatives rather than assumed a priori to represent valid farm types.

Third, archetypal analysis was applied to the retained FAMD dimensions to evaluate continuous reference configurations [31,32]. Candidate solutions from (*k* = 2) to (*k* = 6) archetypes were compared. Reconstruction performance was evaluated using repeated fivefold cross-validation with 20 repetitions, and center stability was assessed using 300 bootstrap resamples for each candidate value of (*k*). Selection considered residual reconstruction error, held-out reconstruction error, marginal improvement relative to simpler solutions, separation among archetypal centers, the presence of farms with mixed affinities, bootstrap reliability, interpretability, and parsimony. Reconstruction error was not used alone: it decreases mechanically as additional archetypes are introduced. In the retained solution, each farm was represented by non-negative affinity coefficients that summed to one, allowing intermediate positions between reference configurations.

The final retained archetype solution was subjected to an additional stability analysis using 1000 bootstrap resamples [33]. Bootstrap archetypal centers were aligned with the original solution using Procrustes transformation and optimal matching. Stability was summarized using dominant-label agreement, correlations between the original and bootstrap affinity coefficients, and archetype-center displacement. Archetype-center displacement was defined as the Euclidean distance, in the standardized two-dimensional FAMD space, between each Procrustes-aligned bootstrap archetype center and its optimally matched center in the original solution; smaller distances indicated greater geometric stability. Archetypal profiles were subsequently backtransformed to the original variable scales to facilitate their substantive interpretation.

Fourth, economic relevance was evaluated using annual net benefit and pasture opportunity cost as independent criteria. These variables were excluded from FAMD and archetype construction to avoid circular validation [25]. Robust regression models were used to examine their relationships with the retained FAMD dimensions and archetypal affinities [34]. Additional sensitivity analyses considered household-head age, ethnicity, and institutional engagement. Institutional engagement was derived from two source-survey items association membership and self-reported active participation and was classified into three categories: non-member, inactive member, and active member. Uncertainty in the economic associations was estimated using 2000 bootstrap resamples [33].

Fifth, predictive performance was evaluated through repeated fivefold cross-validation with 30 repetitions. Six models were compared: a mean-only null model, linear regression based on the retained FAMD dimensions, robust regression based on the FAMD dimensions, robust regression based on archetypal affinities, elastic net, and random forest. A median-only null prediction was also included as an additional benchmark for mean absolute error. Performance was assessed using mean absolute error, root mean squared error, and out-of-sample (*R*^2^). To prevent information leakage, FAMD transformations and archetypal solutions were re-estimated within each training fold before predictions were generated for the corresponding held-out farms. Elastic-net regularization was fitted using internal tuning [35,36], whereas random forests were used to evaluate flexible nonlinear relationships [37]. Permutation-based variable importance was calculated exclusively from held-out observations.

All analyses were conducted in R version 4.6.0. (R Foundation for Statistical Computing, Vienna, Austria) [38]. The FAMD transformation, projection, permutation, and resampling procedures were implemented directly in the reproducibility scripts [28]. The cluster package was used to calculate the Gap statistic and silhouette width [29,30], archetypes for archetypal analysis [31], MASS for robust regression [39], glmnet for elastic-net regression [35], and ranger for random forests [37]. Reproducibility was supported through fixed random seeds and prespecified permutation, bootstraps, and cross-validation procedures.

## 3. Results

### 3.1. Data Structure and Retained Dimensions

**Figure 3:**
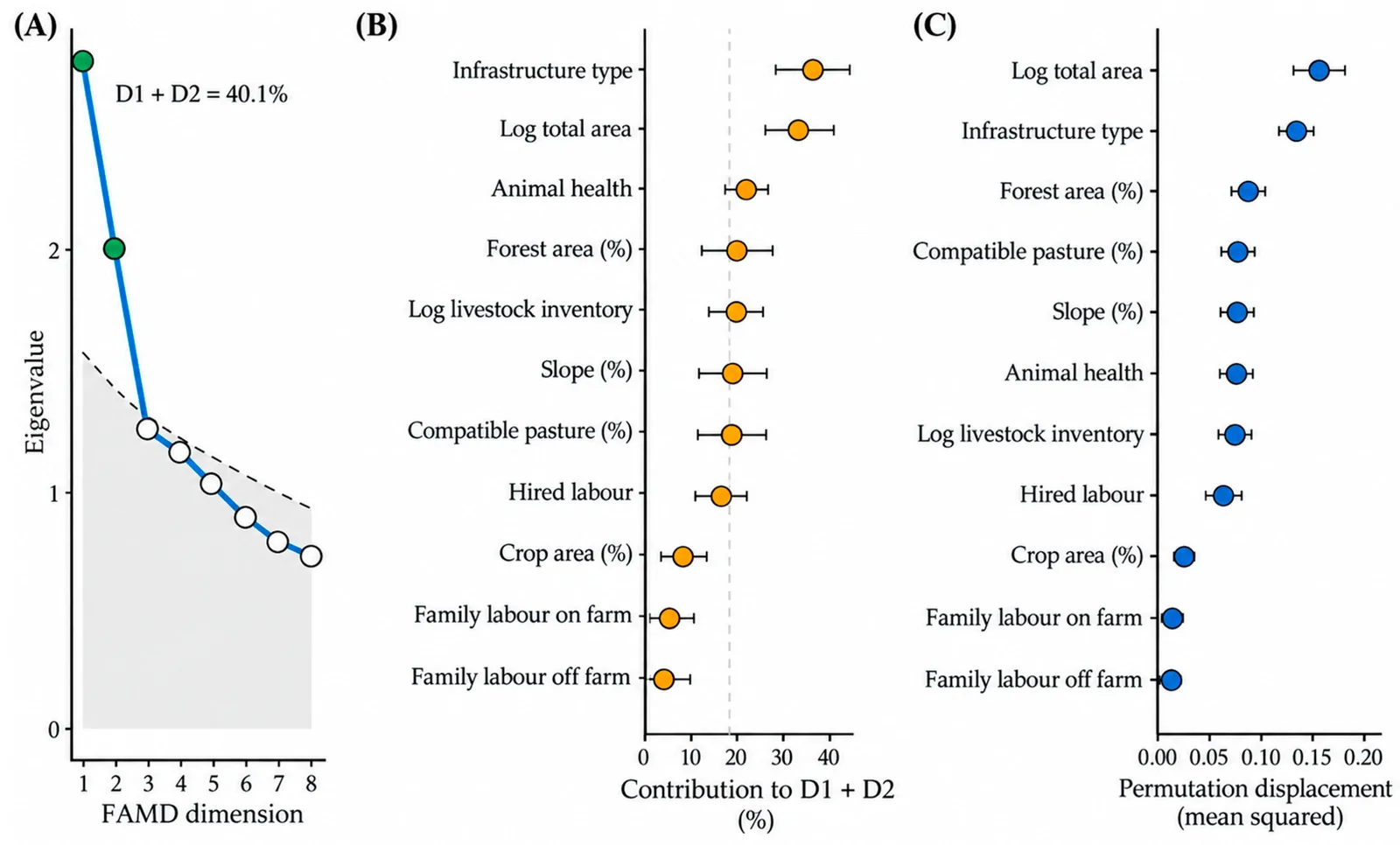
FAMD dimension retention and variable stability. (**A**) Observed eigenvalues (blue line) and the 95th-percentile null threshold obtained from 1000 permutations (black dashed line). The gray shaded area denotes values at or below the null threshold. Green filled circles indicate retained dimensions, whereas open circles indicate non-retained dimensions. (**B**) Contributions to D1 + D2; orange points show bootstrap medians and horizontal lines indicate the 2.5th–97.5th percentiles from 1000 resamples. The vertical dashed line indicates the equal expected contribution (18.18%). (**C**) Permutation displacement, defined as the mean squared change in standardized FAMD scores after independently permuting each variable across farms. Blue points show medians and horizontal lines indicate the 2.5th–97.5th percentiles from 1000 permutations; larger values indicate greater influence on the FAMD representation. Colors in panels (**B**,**C**) are used only to distinguish the panels.

Two FAMD dimensions exceeded the permutation-based null threshold and together explained 40.1% of the mixed-data inertia (Figure 3A). Dimension 1 represented livestock intensification and infrastructure, whereas Dimension 2 reflected farm scale and land use. Infrastructure type and total farm area were the dominant contributors, followed by animal-health, forest cover, livestock inventory, grazing-compatible pasture, and slope. Bootstrap resampling confirmed the stability of these contributions (Figure 3B). Total farm area and infrastructure type also produced the largest permutation displacements, confirming their strong influence on the retained structural representation (Figure 3C). Finally, the multiblock sensitivity analysis yielded an almost identical configuration between FAMD and MFA (full Procrustes (*R*^2^ = 0.990)), indicating that the representation was robust and not driven by differences in block size.

### 3.2. Continuous Livestock Archetypes

**Figure 4:**
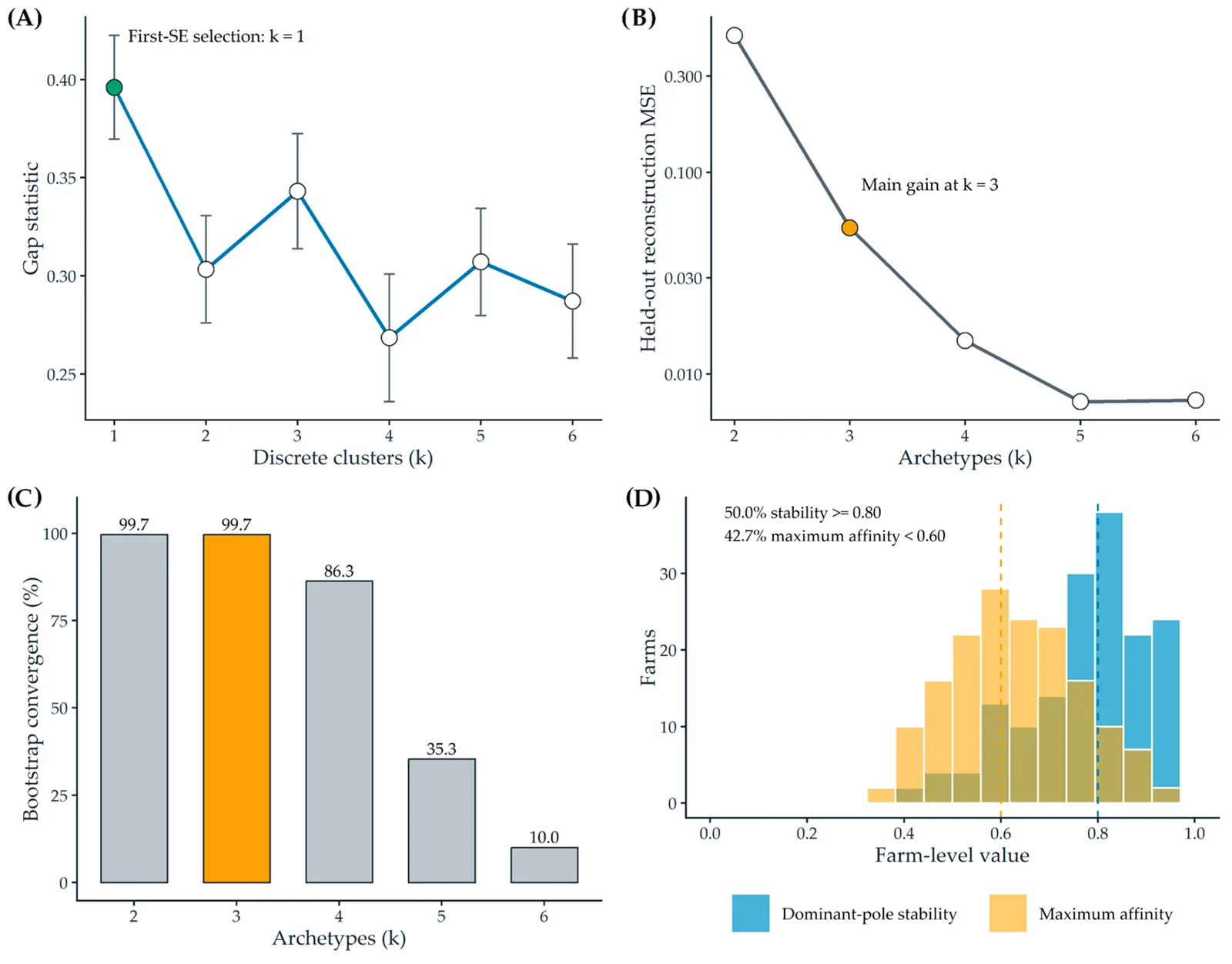
Evidence supporting continuous archetype selection. (**A**) Gap statistic with standard error bars for (*k* = 1)–6; the first-SE rule selected (*k* = 1). (**B**) Median held-out reconstruction error from repeated fivefold cross-validation for (*k* = 2)–6. (**C**) Bootstrap convergence across 300 resamples for each candidate solution; (*k* = 3) is highlighted. (**D**) Distribution of maximum farm affinity; dashed lines indicate mixed affinity (<0.60) and high dominance (≥0.80).

Before interpreting the archetypal solutions, we assessed whether the retained FAMD space supported discrete farm groups. The Gap statistic was highest at (*k* = 1) (*Gap* = 0.396, *SE* = 0.026), and the first standard-error rule also selected (*k* = 1) (Figure 4A). For the forced partitions, mean silhouette widths were 0.398, 0.382, 0.317, 0.359, and 0.372 for (*k* = 2, 3, 4, 5,) and (6), respectively. These values indicated only weak-to-moderate separation and did not provide convergent evidence of reproducible discrete farm boundaries. Accordingly, no mutually exclusive farm typology was retained. Archetypal analysis instead identified three continuous reference configurations that provided the best balance among goodness of fit, out-of-sample reconstruction performance, interpretability, and bootstrap stability (Figure 4B–D). Solutions with four or more archetypes achieved only marginal improvements in reconstruction error but showed lower reproducibility. Farm affinities also revealed extensive overlap among archetypes, with many farms occupying intermediate positions rather than displaying exclusive membership.

### 3.3. Three Reference Configurations Within a Continuum

**Figure 5:**
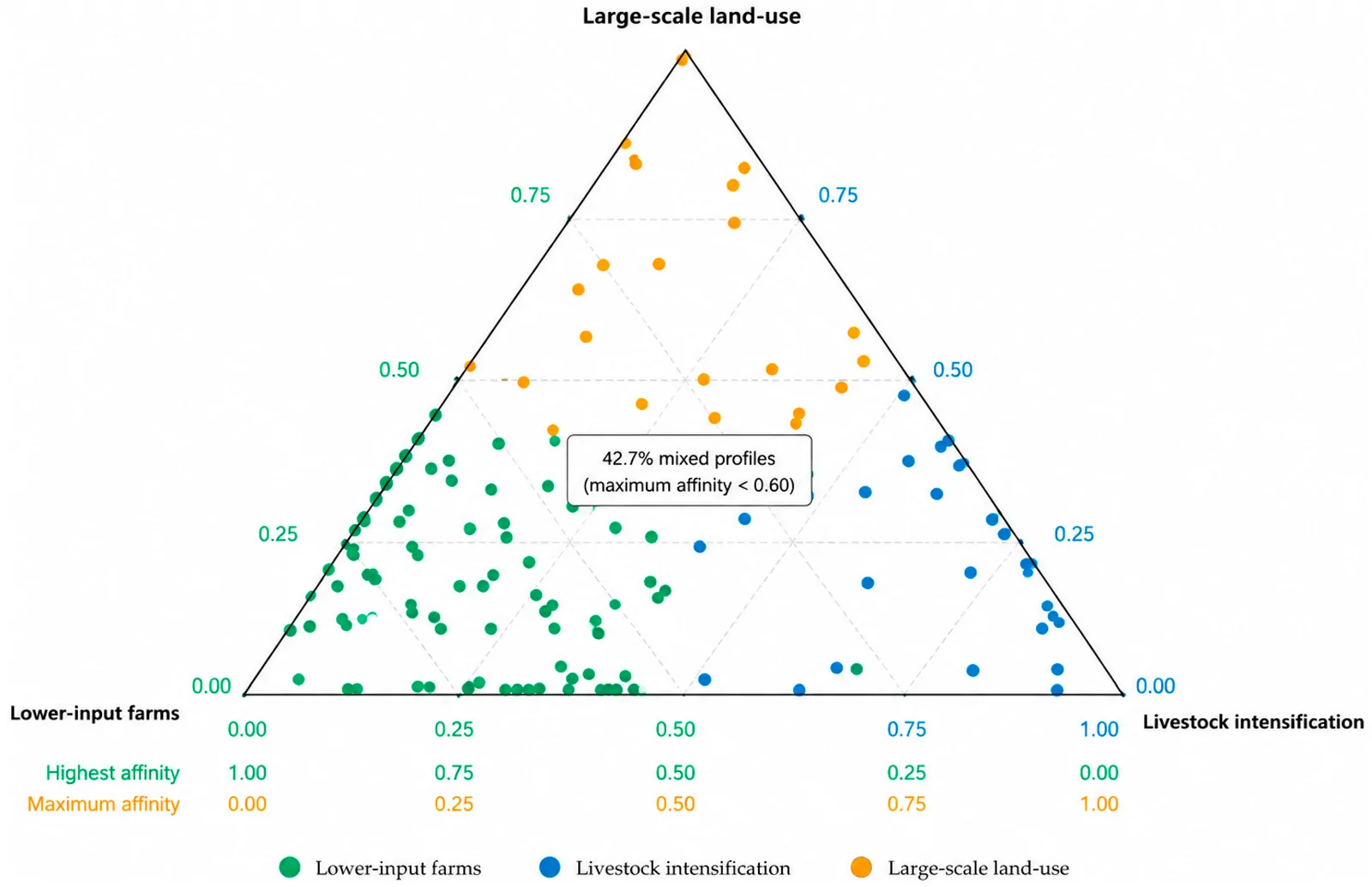
Continuous affinities of 164 farms to the three livestock archetypes. Each vertex represents complete affinity to one reference configuration: lower-input farms (**left**), livestock intensification (**right**), and large-scale land-use (**top**). Points represent farms and are colored according to their highest affinity; interior positions indicate mixed profiles. Light-gray dashed grid lines indicate constant affinity levels at increments of 0.25 for each archetype.

Many farms occupied intermediate positions within the ternary space rather than a single vertex, indicating mixed affinities among the three reference livestock archetypes (Figure 5). The lower-input archetype was the dominant affinity for 72.0% of farms; however, these counts describe proximity to the reference poles and should not be interpreted as mutually exclusive farm groups.

The structural profiles defining the three continuous livestock archetypes are summarized in Table 2. The lower-input archetype was characterized by relatively small farms with few cattle, high forest cover, and little or no productive or animal-health. The livestock intensification archetype combined small farm size with greater stocking levels, a high proportion of grazing-compatible pasture, hired labor, and well-developed productive and animal-health. In contrast, the large-scale land-use archetype was characterized by extensive land holdings, larger herds, high forest cover, and widespread animal-health, while productive infrastructure remained less developed.

Of the 1000 final bootstrap replicates, 998 converged successfully. Median correlations between the original and bootstrap affinity scores were 0.946, 0.941, and 0.921 for the lower-input, livestock intensification, and large-scale land-use archetypes, respectively; the corresponding 95% bootstrap percentile intervals were 0.473–0.996, 0.383–0.996, and 0.416–0.994. Median archetype-center displacements were 0.803, 0.805, and 0.928 standardized FAMD units, with percentile intervals of 0.105–2.066, 0.089–2.449, and 0.099–2.811, respectively. These results indicated high typical agreement between the original and bootstrap solutions, although the interval widths revealed greater uncertainty in some resamples.

### 3.4. Economic Distributions and Robust Associations

**Figure 6:**
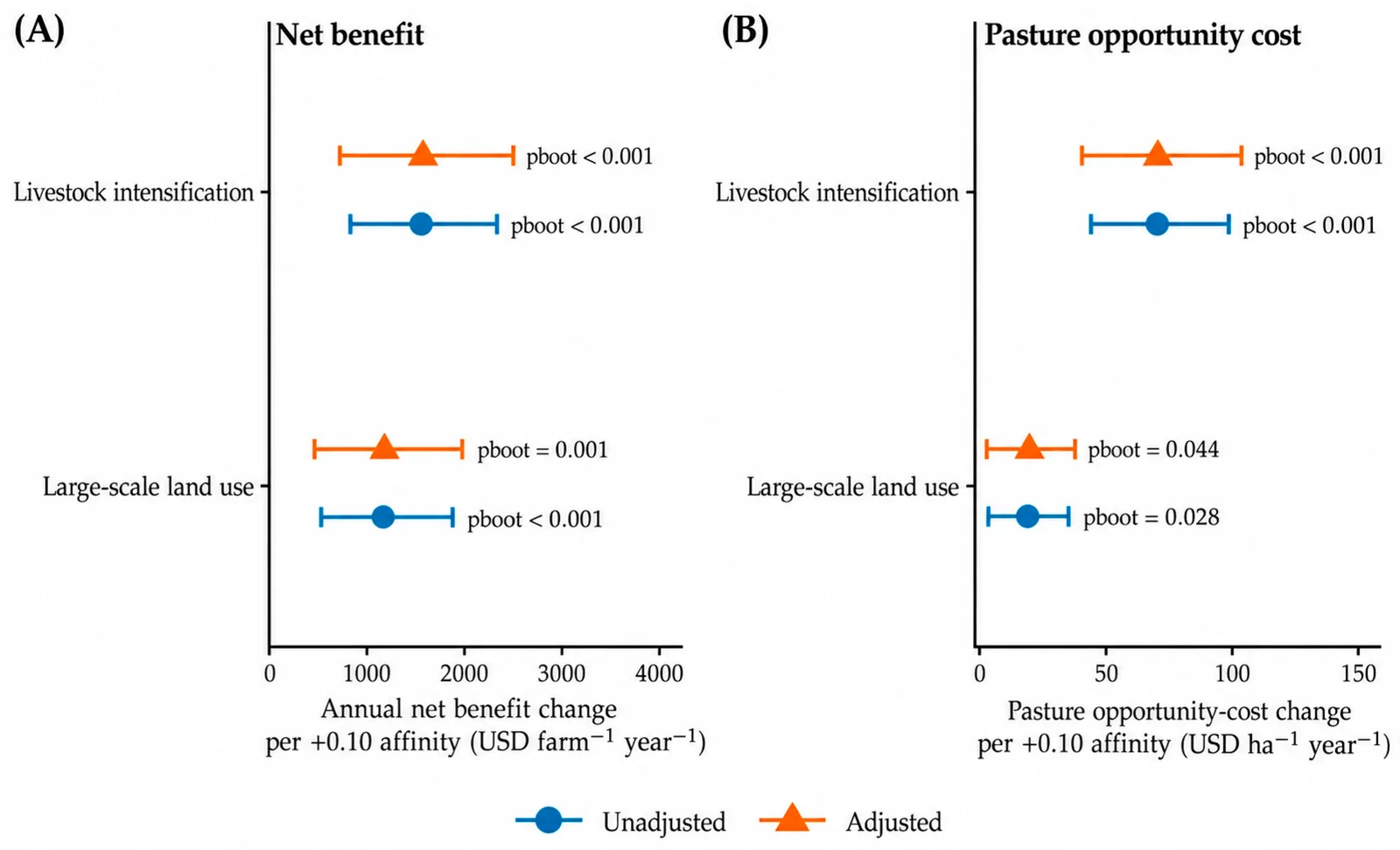
Robust associations of archetype affinities with (**A**) annual net benefit and (**B**) pasture opportunity cost. Estimates represent associations with a +0.10 increase in the displayed affinity and an equivalent decrease in lower-input reference affinity, while holding the third affinity constant; lines indicate 95% bootstrap confidence intervals from 2000 resamples. Adjusted models included age, ethnicity, and institutional engagement (unadjusted: *n* = 163; adjusted: *n* = 161). Associations are descriptive and non-causal.

Annual net benefit and pasture opportunity cost showed markedly right-skewed distributions, with substantial variability among farms and numerous negative values for both outcomes. These characteristics supported the use of robust regression models. Both the FAMD dimensions and the continuous livestock archetype affinities were associated with the evaluated economic outcomes (Figure 6). Detailed robust regression results are reported in Supplementary Table S1. Within the FAMD representation, the livestock intensification and infrastructure dimension showed consistent positive associations with both economic outcomes. In contrast, the farm scale and land-use dimension showed no consistent association with annual net benefit but was negatively associated with pasture opportunity cost.

In the affinity-based models, lower-input affinity was used as the omitted reference because the three affinity scores of each farm sum to one. Accordingly, Figure 6 presents only the contrasts for livestock intensification and large-scale land-use. Each estimate represents the association corresponding to a +0.10 increase in the displayed affinity and an equivalent decrease in lower-input affinity, while holding the third affinity constant. Relative to this reference, livestock intensification affinity showed the strongest positive associations with both economic outcomes, whereas large-scale land-use affinity showed smaller positive associations. The estimates remained largely unchanged after adjustment for age, ethnicity, and institutional engagement, while the additional covariates showed no consistent associations with either outcome. Affinity-weighted summaries were consistent with these results: economic performance was lowest toward the lower-input configuration, highest toward livestock intensification, and intermediate toward the large-scale land-use configuration.

### 3.5. Predictive Performance

Repeated fivefold cross-validation showed that the robust archetype model achieved the lowest median MAE for both annual net benefit and pasture opportunity cost compared with the mean-null, FAMD2 linear, FAMD2 robust, elastic-net, and random-forest models. Its prediction errors were also lower than those of the median-null benchmark for both outcomes. However, the archetype model did not consistently minimize RMSE or maximize out-of-sample (*R*^2^): random forest achieved the highest (*R*^2^) for net benefit, whereas FAMD2 linear regression achieved the highest value for pasture opportunity cost. Overall, predictive performance remained modest, indicating that the archetype framework is better suited to structural interpretation and differentiated management guidance than to precise economic prediction. Complete cross-validation results are reported in Supplementary Table S2.

**Figure 7:**
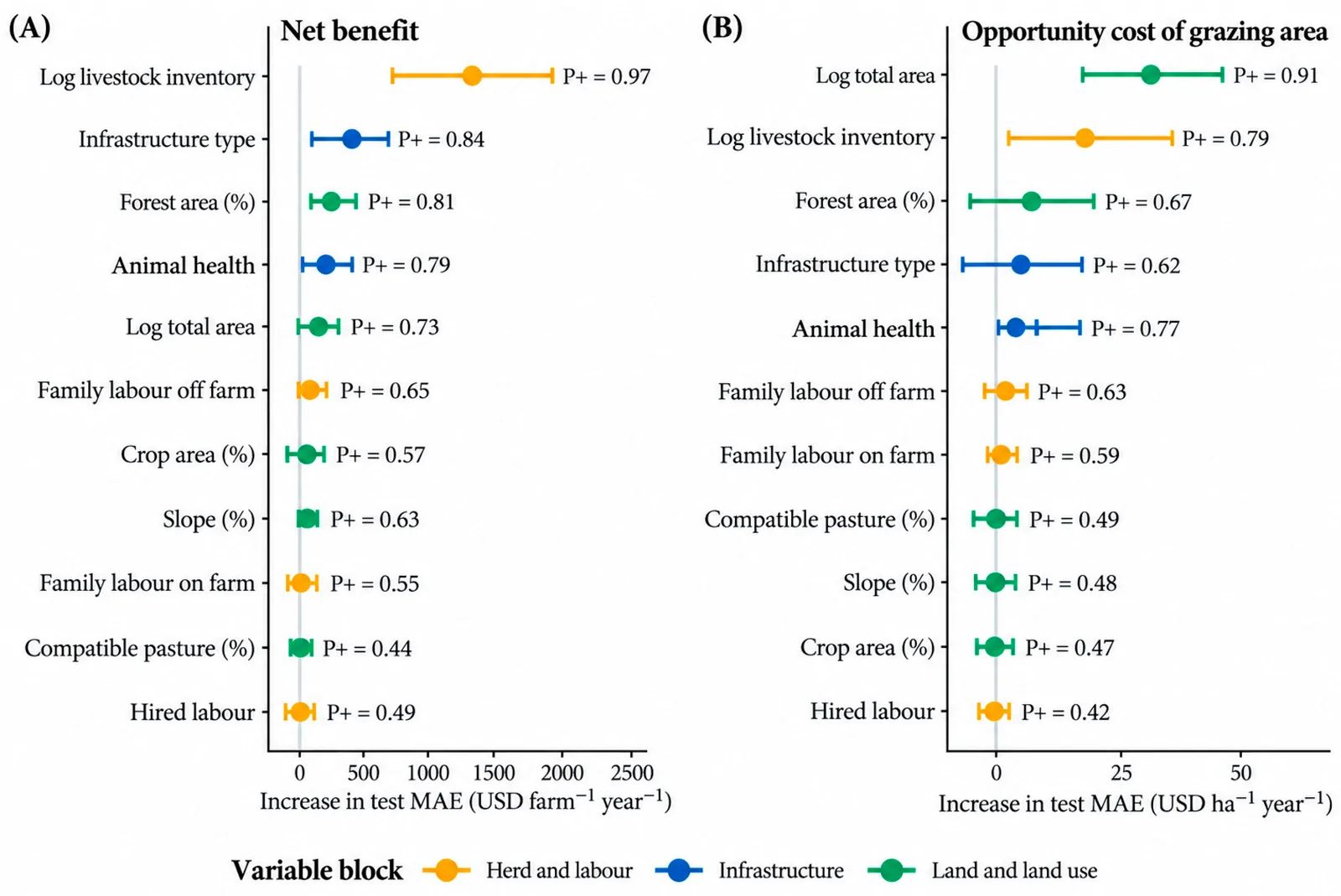
Out-of-sample random-forest permutation importance for (**A**) annual net benefit and (**B**) pasture opportunity cost. Points show the median increase in held-out MAE after permuting each variable, and bars show the interquartile range across folds. The gray vertical line indicates zero change in held-out MAE. Colors identify conceptual blocks. P+ is the proportion of held-out folds with increased MAE and is not a *p*-value; larger positive values indicate greater predictive importance.

Out-of-sample random-forest permutation importance identified livestock inventory as the strongest predictor of net benefit, followed by infrastructure type, forest cover, animal-health, and farm area (Figure 7A). Farm area was the strongest predictor of pasture opportunity cost, followed by livestock inventory and forest cover (Figure 7B). At the block level, the herd-and-labor block, driven primarily by livestock inventory, contributed most to net-benefit prediction, whereas the land-and-land-use block, driven primarily by total farm area, was the most influential predictor of pasture opportunity cost.

## 4. Discussion

### 4.1. From Farm Classes to Continuous Livestock Configurations

The present study shows that the structural heterogeneity of dual-purpose cattle farms in the Ecuadorian Amazon is effectively represented by three continuous livestock archetypes. Rather than defining mutually exclusive farm classes, these archetypes describe farms as continuous combinations of reference structural configurations. This representation better reflects the diversity of smallholder livestock systems, where farms often combine characteristics associated with different production strategies. Three reference configurations summarized the main directions of structural variation without requiring discrete boundaries between farms.

These findings are consistent with the original concept of archetypal analysis, which represents observations as mixtures of extreme reference configurations rather than assigning them to average cluster centres. Similar advantages have been reported in agricultural systems, where continuous archetypes capture structural variation that may remain hidden under conventional categorical typologies [8]. The high stability of the continuous affinity scores across bootstrap samples further supports the robustness of this representation.

Cluster-based approaches remain valuable when well-defined groups are supported by the data [11,13]. However, farming systems are increasingly recognized as dynamic configurations that evolve over time rather than fixed structural categories [14]. Under these conditions, continuous livestock archetypes provide a more flexible framework for describing farm heterogeneity and supporting tailored management interventions.

The two retained FAMD dimensions explain the structural basis of these archetypes. The first dimension represented livestock and technological capacity, integrating herd size, productive infrastructure, and animal-health facilities. The second represented territorial scale and land use, mainly reflecting farm area and forest cover. This separation indicates that technological development and territorial resources are distinct structural capacities rather than different expressions of the same production gradient. Similar relationships have been described in tropical livestock systems, where farm performance depends on combinations of structural resources, technology, and land use rather than farm size alone [6,25].

Together, these structural capacities define three complementary livestock archetypes rather than successive stages of development. The lower-input archetype reflects limited technological resources and infrastructure. The livestock intensification archetype combines relatively high productive capacity within a limited land base. The large-scale land-use archetype represents greater territorial resources and forest availability without necessarily achieving the highest economic returns per hectare. These continuous configurations provide a practical framework for tailoring management interventions according to the structural characteristics and opportunities of each farm.

### 4.2. Economic Differentiation Supports Tailored Management Interventions

The continuous livestock archetypes identified in this study provided a practical framework for economically differentiating dual-purpose cattle farms and supporting tailored management interventions. Farms with greater affinity to the livestock intensification archetype consistently showed the strongest economic performance, whereas the large-scale land-use archetype occupied an intermediate position. The FAMD representation supported the same interpretation, indicating that livestock and technological capacity contributed more strongly to economic performance than territorial scale alone. Together, these findings demonstrate that structural farm profiles capture economically meaningful differences that can guide differentiated management strategies.

These associations should be interpreted as relationships rather than causal effects. Farm performance reflects the interaction of structural resources, management, market conditions, and previous investments. Consequently, the archetypes represent structural potential rather than direct determinants of profitability. This interpretation agrees with previous studies showing that farm performance depends on the combined effects of technological, structural, and management factors rather than on any single characteristic [25].

The predictive analysis further reinforces the practical value of the proposed framework. Although predictive performance remained modest, the archetype-based models consistently reduced prediction error compared with the resistant baseline and performed as well as or better than more complex machine learning approaches under the mean absolute error criterion. Similar frameworks have been proposed to support strategic planning and farm characterization rather than to automate management decisions [40]. Continuous livestock archetypes should therefore be used as a first decision-support layer for screening farms and prioritizing tailored management interventions. Farm-level recommendations should subsequently incorporate additional information on production, costs, management practices, and market conditions before investment or policy decisions are made.

### 4.3. Practical Implications for Tailored Management Interventions

The continuous livestock archetypes identified in this study provide a practical framework for tailoring management interventions in dual-purpose cattle farms. Rather than assigning farms to mutually exclusive classes, the proposed framework captures structural heterogeneity through continuous affinities, allowing management recommendations to be adapted to the characteristics of each farm.

The present study shows that the structural heterogeneity of dual-purpose cattle farms in the Ecuadorian Amazon is better represented by continuous livestock archetypes than by mutually exclusive farm classes. Three reference configurations summarized the main structural variation while allowing farms to combine different characteristics according to their affinity scores. This continuous representation better reflects the complexity of tropical livestock systems and provides a more flexible framework for farm characterization than conventional typologies [5]. Cluster-based approaches remain useful when clear group boundaries exist [11,13], but continuous archetypes are particularly appropriate when structural variation follows gradients rather than discrete categories [14].

The two structural dimensions identified by FAMD further support this interpretation. Livestock and technological capacity were clearly separated from territorial scale and land use, indicating that farm size alone is not a reliable proxy for production intensity. Similar relationships have been described in tropical livestock systems, where productivity depends on the interaction between structural resources, technology, and management rather than on any single characteristic [6,25,41].

Beyond their structural interpretation, the continuous livestock archetypes also differentiated farms economically. Greater affinity to the livestock intensification archetype was associated with higher economic performance, whereas the large-scale land-use archetype showed intermediate outcomes. These relationships remained consistent after adjustment for household characteristics, suggesting that the archetypes capture economically meaningful differences among farms. Nevertheless, they should be interpreted as structural associations rather than causal relationships, since farm performance also depends on management decisions, market conditions, and previous investments [25].

Recent bibliometric evidence shows that agroforestry research integrates productive, environmental, climatic, and socioeconomic dimensions, with rural livelihoods embedded within a broader sustainability-oriented structure [42]. The present results complement this evidence at the farm level: the three continuous archetypes represented different combinations of livestock, labor, infrastructure, land use, and forest resources, while independent economic outcomes differentiated their affinities. However, biodiversity, climate, and comprehensive livelihood outcomes require specific indicators and further validation.

The practical value of the proposed framework lies in translating structural heterogeneity into differentiated management interventions [40]. Rather than prescribing a single development pathway, the archetypes identify potential starting conditions for technical assistance, restoration planning, and possible silvopastoral transitions [43,44]. Lower-input farms may benefit from gradual improvements in animal health, water management, pasture organization, and record keeping before major investments [40]. Farms with stronger livestock intensification affinity may be better suited to interventions that improve productivity per animal and per hectare through grazing management, forage resources, shade, and animal health [44,45,46]. In contrast, the large-scale land-use archetype may provide suitable conditions for integrating livestock production with ecological restoration, riparian protection, and forest conservation [47,48], particularly where pasture opportunity costs are lower [21]. The predictive analysis reinforces this interpretation. Although predictive accuracy remained modest, archetype-based models consistently reduced prediction error compared with resistant baseline models and performed competitively with more complex machine learning approaches. Similar frameworks have been proposed as decision-support tools for strategic planning rather than for automated decision making [40]. Continuous livestock archetypes should therefore be viewed as a first screening layer that supports tailored management interventions. Farm-level recommendations should subsequently incorporate current production, economic, environmental, and social information before investment or policy decisions are implemented.

Finally, the present study extends previous analyses conducted in the Sumaco Biosphere Reserve by moving beyond predefined altitudinal classifications [20,21]. Instead of grouping farms according to environmental strata, the proposed framework characterizes structural heterogeneity directly, validates continuous livestock archetypes through independent economic criteria, and translates these profiles into differentiated management recommendations. This evidence-first approach provides a more flexible basis for supporting sustainable livestock development under heterogeneous production conditions.

The following evidence-to-action matrix distinguishes findings from the present study from literature-informed management options (Table 3). The proposed actions were not experimentally tested and should therefore be treated as conditional orientations requiring current farm- and parcel-level assessment. Farms with intermediate affinities should not be assigned to a single pathway; their complete affinity profiles should be used to combine and sequence actions from the relevant reference configurations.

### 4.4. Limitations and Strengths

This study combined several complementary validation procedures to develop and evaluate continuous livestock archetypes. Structural dimensions were retained using permutation-based criteria, discrete boundaries were formally tested before archetypes were interpreted, and candidate solutions were evaluated through bootstrap stability, cross-validation, and independent economic validation. Separating structural and economic variables throughout the analytical workflow reduced circularity and strengthened the interpretation of the resulting archetypes.

Several limitations should nevertheless be acknowledged. First, the cross-sectional design does not allow causal inference or assessment of temporal transitions between structural configurations. Second, the study was conducted within a single biosphere reserve, and external validation in other tropical livestock systems is required to evaluate the generality of the proposed framework. Third, farm performance is influenced by variables not included in the structural characterization, including production efficiency, market dynamics, and management decisions. Family-labor variables represented counts of household members rather than labor intensity because working hours, annual workdays, participation duration, and full-time equivalents were not recorded. The database also lacked a detailed inventory of machinery and technical equipment, limiting the assessment of farm technological capacity and development potential. Fourth, the source survey did not include a validated, multidimensional animal welfare assessment, nor did it capture direct indicators such as body condition, lameness, injuries, behavioral indicators, morbidity, or thermal stress. The variable evaluating housing conditions used as a proxy for animal health and welfare was based solely on the physical rearing environment and the space available per animal; therefore, it should not be interpreted as a comprehensive measure of overall animal welfare. Furthermore, because this classification relied on qualitative field assessments rather than predefined quantitative thresholds, its reproducibility across independent survey teams may be limited. Finally, the database did not include detailed information on tree composition, silvopastoral design, or ecosystem-service indicators, preventing the identification of specifically silvopastoral archetypes.

Future research should evaluate the temporal stability of continuous livestock archetypes, validate the framework in other tropical production systems, and integrate productive, environmental, social, labor-intensity, technical-equipment, and animal-welfare indicators within longitudinal analyses. Combining continuous structural archetypes with detailed information on technological capacity, validated welfare measures, and silvopastoral design would provide an important step toward supporting adaptive livestock management under increasingly complex environmental and socioeconomic conditions.

## 5. Conclusions

The evidence-first analytical workflow provided no convergent evidence supporting mutually exclusive farm groups in the study population. Instead, the combined use of FAMD, permutation-based dimension retention, clusterability assessment, archetypal analysis, bootstrap resampling, cross-validation, and independent economic criteria supported a continuous representation of structural heterogeneity. Further research should evaluate the reproducibility and external validity of this framework in other smallholder dual-purpose cattle systems under comparable tropical and socioeconomic conditions.

Three continuous archetypes summarized the principal structural configurations of cattle farms in the Sumaco Biosphere Reserve: lower-input, livestock intensification, and large-scale land-use. These reference configurations represented different combinations of livestock inventory, infrastructure, labor, land use, and forest resources. The affinity-based representation allowed farms to occupy intermediate positions and express characteristics of more than one configuration without being assigned to mutually exclusive types. Greater affinity to livestock intensification was associated with more favorable economic outcomes, whereas the large-scale land-use configuration showed intermediate economic performance. These associations should not be interpreted as causal or as evidence of temporal development pathways.

The archetypes therefore provide a screening and decision-support framework for formulating differentiated literature-informed management options. These may include gradual improvements in animal health, water management, grazing organization, and farm records for lower-input profiles; improvements in productivity per animal and per hectare for farms with stronger livestock intensification affinity; and forest conservation, natural regeneration, riparian protection, and targeted silvopastoral restoration for farms with stronger large-scale land-use affinity. For farms with mixed affinities, actions should be combined and prioritized according to the complete affinity profile. These options were not experimentally tested; therefore, their implementation should be guided by current farm- and parcel-level assessments, producer objectives, available resources, and safeguards against deforestation.

## Figures and Tables

**Table 1:** Active variables, transformations, conceptual blocks, and analytical roles. The structural cohort comprised 164 farms; the economic cohort comprised 163 farms.

Analytical Variable	Description
Log total area	Log-transformed total farm area (ha).
Slope, %	Average slope of the farm (%).
Crop area, %	Percentage of farm area under crops.
Forest area, %	Percentage of farm areas covered by forest.
Log livestock inventory	Log-transformed total cattle inventory.
Grazing pasture, %	Percentage of land suitable for grazing.
Family labor on-farm, n	Number of family members working on farm.
Family labor off-farm, n	Number of family members working off farm.
Hired labor	Use of hired labor on the farm. Categories: Yes and No.
Productive infrastructure type	Field-based classification of livestock infrastructure as typical, rustic, or none. None indicated the absence of dedicated livestock infrastructure. Rustic indicated basic facilities, generally limited to a simple stable built mainly with locally available farm materials and without specific technical design. Typical indicated improved and more developed facilities, such as stables, storage facilities, and sheds, with greater built area and functional capacity. Classification was based on the assessment of the trained interviewer familiar with local livestock systems.
Animal health and welfare housing conditions	Binary field-based assessment of whether the farm provided housing and rearing conditions considered appropriate for animal health and welfare, based on the rearing environment and space available per animal. Categories were Yes and No. Classification was based on the assessment of the trained interviewer familiar with local livestock systems.

Note: Family-labor variables are person counts because work-time data were unavailable.

**Table 2:** Structural profiles of the three continuous livestock archetypes.

Indicator	Lower-Input Farms	Livestock Intensification	Large-Scale Land-Use
Total area, ha	29	10	210
Livestock inventory, head	6	12	35
Forest area, %	55.2	0	76.2
Grazing-compatible pasture, %	85	95	40
Hired labor, %	0	99.4	100
Animal-health, %	0	100	82.7

Note: Values are back-transformed archetypal coordinates representing the reference configurations and should not be interpreted as sample means or summaries of mutually exclusive farm groups.

**Table 3:** Evidence-to-action-user-safeguard matrix for differentiated management of dual-purpose cattle farms.

Evidence from the Study	Conditional Management Option	Main Users	Key Safeguard
Lower-input farms combined smaller herds, limited infrastructure, and lower economic performance.	Prioritize animal health, water and grazing management, record keeping, and gradual silvopastoral improvements [49,50].	Extension services, veterinarians, producer organizations.	Adapt actions to farm objectives and available resources.
Livestock-intensification affinity showed greater livestock and infrastructure endowment and the strongest economic relationships.	Improve productivity per animal and hectare through grazing, forage trees, shade, and animal-health management [45,51].	Farmers, livestock advisers, silvopastoral technicians.	Avoid deforestation and monitor stocking rate, pasture condition, costs, and welfare.
Large-scale land-use farms combined extensive holdings, high forest cover, and intermediate economic outcomes.	Integrate livestock production with trees, riparian protection, natural regeneration, and restoration where opportunity costs are lower [21,47,48].	Conservation agencies, reserve managers, local governments, landholders.	Base actions on parcel-level conditions and opportunity costs.
Archetype affinities supported structural interpretation, but economic prediction remained modest.	Use archetypes as a screening tool for differentiated technical assistance [2,40].	Extension planners, researchers, policy makers.	Do not use archetypes alone for credit, subsidies, or investment decisions.
Many farms occupied intermediate positions; 42.7% had maximum affinity < 0.60.	Use the complete affinity profile to combine and sequence actions from relevant configurations [2,14].	Farmers, researchers, extension services.	Avoid permanent labels and reassess priorities as farm conditions change.

Note: The first column reports findings from this study. Management options are literature-informed, conditional recommendations and were not experimentally tested.

## Data Availability

The data used in this study are not deposited in a publicly accessible repository, as they contain confidential information at the household and farm levels and are subject to the data governance rules of the original research. Selected data and reproducible analysis scripts may be made available upon reasonable request to the data controller (btorres@uea.edu.ec) and subject to applicable confidentiality and governance conditions.

## Supplementary Materials

The following supporting information can be downloaded at https://www.mdpi.com/article/10.3390/ani16182931/s1, Table S1: Robust economic associations; Table S2: Repeated five-fold cross-validation 30 repetitions.
