# Supplementary material for: Continuous Livestock Archetypes for Characterizing Structural Heterogeneity in Dual-Purpose Cattle Farms in the Ecuadorian Amazon
Source: Animals (Basel). 2026 Sep 18;16(18):2931. doi: 10.3390/ani16182931 (PMC13603300; doi:10.3390/ani16182931)
Supplement: Supplementary file 1 [file animals-16-02931-s001.zip › animals-4523151-supplementary/Supplementary_Table_S1_Robust_economic_associations.pdf]

Supplementary Material

Continuous Livestock Archetypes: A Strategic Framework for Tailored Management Interventions in Dual-Purpose Cattle Farms in the Ecuadorian Amazon

Table S1. Robust economic associations.

Outcome	Representation	Specification	n	Term	Estimate	95% bootstrap CI	pboot
Net benefit	FAMD	Unadjusted	163	Livestock intensity and infrastructure (SD)	3,870.9	[2,344.2, 5,894.0]	<0.001
Net benefit	FAMD	Unadjusted	163	Farm scale and land use (SD)	475.8	[-345.5, 1,368.7]	0.268
Net benefit	FAMD	Adjusted	161	Livestock intensity and infrastructure (SD)	4,003.9	[2,073.2, 6,799.3]	<0.001
Net benefit	FAMD	Adjusted	161	Farm scale and land use (SD)	493.0	[-423.8, 1,466.4]	0.280
Net benefit	Archetype	Unadjusted	163	Livestock intensification affinity	1,669.4	[1,015.3, 2,526.5]	<0.001
Net benefit	Archetype	Unadjusted	163	Large scale land use affinity	1,215.1	[622.5, 1,986.1]	<0.001
Net benefit	Archetype	Adjusted	161	Livestock intensification affinity	1,743.5	[905.1, 2,836.4]	<0.001
Net benefit	Archetype	Adjusted	161	Large scale land use affinity	1,279.6	[592.6, 2,126.6]	0.001
Opportunity cost of grazing area	FAMD	Unadjusted	163	Livestock intensity and infrastructure (SD)	136.2	[89.9, 188.0]	<0.001
Opportunity cost of grazing area	FAMD	Unadjusted	163	Farm scale and land use (SD)	-50.5	[-92.5, -12.5]	0.007
Opportunity cost of grazing area	FAMD	Adjusted	161	Livestock intensity and infrastructure (SD)	136.1	[80.3, 217.9]	<0.001
Opportunity cost of grazing area	FAMD	Adjusted	161	Farm scale and land use (SD)	-47.7	[-94.8, -9.9]	0.011
Opportunity cost of grazing area	Archetype	Unadjusted	163	Livestock intensification affinity	68.6	[49.3, 94.7]	<0.001
Opportunity cost of grazing area	Archetype	Unadjusted	163	Large scale land use affinity	19.2	[2.1, 33.5]	0.028
Opportunity cost of grazing area	Archetype	Adjusted	161	Livestock intensification affinity	69.1	[45.7, 109.8]	<0.001
Opportunity cost of grazing area	Archetype	Adjusted	161	Large scale land use affinity	19.8	[0.8, 39.3]	0.044
