# Supplementary material for: Continuous Livestock Archetypes for Characterizing Structural Heterogeneity in Dual-Purpose Cattle Farms in the Ecuadorian Amazon
Source: Animals (Basel). 2026 Sep 18;16(18):2931. doi: 10.3390/ani16182931 (PMC13603300; doi:10.3390/ani16182931)
Supplement: Supplementary file 1 [file animals-16-02931-s001.zip › animals-4523151-supplementary/Supplementary_Table_S2_Repeated_five-fold_cross-validation_30_repetitions.pdf]

Supplementary Material

Continuous Livestock Archetypes: A Strategic Framework for Tailored Management Interventions in Dual-Purpose Cattle Farms in the Ecuadorian Amazon

Table S2. Repeated five-fold cross-validation, 30 repetitions.

Outcome	Model	Median MAE	Median CV R^2	MAE gain vs median null (%)
Net benefit	Archetype robust	5,580.5	0.126	11.8
Net benefit	FAMD2 robust	5,653.3	0.128	10.7
Net benefit	Random forest	5,916.5	0.160	6.5
Net benefit	Null median (MAE benchmark)	6,327.9	-0.081	0.0
Net benefit	FAMD2 linear	6,368.6	0.147	-0.6
Net benefit	Elastic net	7,164.0	0.025	-13.2
Net benefit	Null mean	7,429.3	-0.011	-17.4
Opportunity cost of grazing area	Archetype robust	256.8	0.081	12.4
Opportunity cost of grazing area	FAMD2 robust	261.3	0.073	10.8
Opportunity cost of grazing area	Null median (MAE benchmark)	293.0	-0.058	0.0
Opportunity cost of grazing area	Random forest	296.0	0.060	-1.0
Opportunity cost of grazing area	FAMD2 linear	296.9	0.091	-1.3
Opportunity cost of grazing area	Elastic net	325.9	-0.007	-11.2
Opportunity cost of grazing area	Null mean	328.9	-0.013	-12.2

Notes: Association intervals are percentile bootstrap confidence intervals. MAE, mean absolute error; CV, cross-validation; R^2, coefficient of determination. The median null is an additional resistant MAE benchmark
